# The Mean Single Molecule Rate (mSMR) in the Analysis of Fluorescence Fluctuations: Measurements on DNA Mixtures of Defined Composition

**DOI:** 10.1007/s10895-021-02803-3

**Published:** 2021-09-16

**Authors:** Lorenz T. Sparrenberg, Benjamin Greiner, Harald P. Mathis

**Affiliations:** 1 Institute of Biotechnology, RWTH Aachen University, Worringerweg 3, 52074 Aachen, Germany; 2 Fraunhofer Institute for Applied Information Technology FIT, Schloss Birlinghoven 1, 53757 Sankt Augustin, Germany

**Keywords:** Fluorescence fluctuation spectroscopy, Cumulant analysis, Single molecule brightness, Nucleic acids, Diffusion

## Abstract

We present a method for the evaluation of fluorescence fluctuations on the basis of Mandel’s Q parameter, using sampling time-dependent factorial cumulants. By relating the Q parameter to the sampling time, we obtain the *mean single molecule rate* (mSMR), an easy to interpret expression that provides both brightness and diffusion information. The model is suitable for the widely used confocal setups with single photon excitation and a single detection channel. We present a way to correct the mSMR for afterpulsing, dead time and background noise. To account for photokinetic effects at short sampling times, we expand the model by a simple on/off isomerization term, which is similar to the well-known triplet model. The functionality of the mSMR is shown using Monte Carlo simulations. The correction mechanisms and the experimental applicability of the model are then demonstrated by DNA measurements of defined composition. By systematically analyzing DNA mixtures, we can show that at large sampling times, the mSMR correctly describes the single molecule brightness rates and the diffusive properties of DNA molecules. At short sampling times, the photokinetic effects of isomerization are accurately described by the mSMR model. Since additionally the mSMR can easily be corrected for measurement artefacts such as detector dead time, afterpulsing and background noise, this is a valuable advantage over the standard method of fluorescence correlation spectroscopy.

## Introduction

Fluorescence fluctuation spectroscopy (FFS) is an important tool to study biomolecules in solution. It is based on the statistical analysis of fluorescence intensity fluctuations due to the diffusion of fluorescent particles through an excitation volume. The fluorescent or fluorescence-labeled particles are excited by a continuous or pulsed laser. A detector unit captures a fraction of the emitted photons over time. A variety of analytical methods are available for the evaluation of the fluorescence traces. Certainly the most widely used technique is fluorescence correlation spectroscopy (FCS), first described by Magde et al. [1, 2]. It was considerably improved by the introduction of confocal optics, which significantly increased the signal-to-noise ratio up to the single molecule level [3]. Today there is a wide range of applications based on the fundamental concepts of FFS. These methods fall into one of two categories. The methods in the first category investigate fluctuations in the time domain. They include, among others, fluorescence correlation spectroscopy (FCS) and its variants such as higher order FCS (HOFCS) [4–6], fluorescence cross-correlation spectroscopy (FCCS) [3, 7], and fluorescence life time spectroscopy (FLCS) [8, 9]. Methods that investigate fluctuations in the amplitude domain are in the second category. They include, among others, the photon counting histogram (PCH) [10], the fluorescence-intensity distribution analysis (FIDA) [11] and the related fluorescence cumulant analysis (FCA) [12]. Both FIDA and FCA have been extended to larger sampling times, now dubbed fluorescence intensity multi distribution analysis (FIMDA) [13] and time integrated fluorescence cumulant analysis (TIFCA) [14]. Finally, Scales and Swain developed the correlated photon counting histogram (cPCH) for an excitation with two excitation sources and two detection channels and showed that the two categories of FFS methods based on fluctuations in the time domain and on fluctuations in the amplitude domain, respectively, can be unified in one theory [15].

In this paper, we derive a sampling time-dependent model for the analysis of the single molecule brightness based on Mandel’s Q parameter. We start with the first two factorial cumulants as used in FIMDA and TIFCA to calculate the Q parameter for increasing sampling times. We normalize the Q parameter to the sampling time and obtain the *mean single molecule rate* (mSMR) that provides both brightness and diffusion information. The mSMR is suitable for single photon excitation with single channel detection, widely used today in fluorescence correlation spectroscopy. We evaluate this model using Monte Carlo simulations. Then we apply the model to real measured data. Since in real experiments detector artefacts occur in setups with a single laser source and a single detection channel, the measurement data are corrected for the most frequent effects, afterpulsing, detector dead time and background noise. We show that this correction is sufficient for an accurate representation of the data. Finally, we introduce an on/off isomerization term to account for the influence of photokinetic effects at short sampling times in DNA measurements. The simple on/off isomerization term is similar to a triplet term used in other FFS studies. For the measurements under real conditions, we use DNA mixtures of known composition and show that the results of the mSMR analysis provide accurate results that are consistent with the literature. Especially with short sampling times, mSMR is well suited for the analysis of photokinetic effects. The primary aim of this paper is to introduce the mSMR model and show its applicability. Further experimental analyses are beyond the scope of this paper and will be addressed in future publications.

## Theory

We start with the general definition of Mandel’s Q parameter [10, 16],

1 $$\begin{aligned} Q = \frac{\langle \Delta k^2 \rangle - \langle k \rangle }{\langle k \rangle } = \frac{\langle k^2 \rangle -\langle k \rangle ^2-\langle k \rangle }{\langle k \rangle } \end{aligned}$$

where $\langle \dots \rangle$ is a time average and denotes the moments of the photon counts *k*. The Q parameter is a measure of the deviation of a photon number from a Poisson distribution for which $Q = 0$ [16]. Q parameters greater than zero are called superpoissonian and Q parameters less than zero are called subpoissonian. The relevance of Mandel’s Q parameter for correlation analyses of diffusing molecules has already been reported in other studies [17, 18]. We can express the first and second ordinary moments of the photon counts *k* in terms of intensity cumulants [19, 20] and get:

2 $$\begin{aligned} Q = \frac{\kappa _{2}}{\kappa _{1}}, \end{aligned}$$

with the intensity cumulants $\kappa _{1} = \langle k \rangle$ and $\kappa _{2} = \langle k^2 \rangle - \langle k \rangle ^2 - \langle k \rangle$. For short sampling times compared to the characteristic diffusion time $\tau _D$ of the molecules through the observation volume, the first two intensity cumulants are given by [17]:

3 $$\begin{aligned} \kappa _{1}&= \epsilon \,N,\end{aligned}$$

4 $$\begin{aligned} \kappa _{2}&= \gamma _2\,\epsilon ^2\,N, \end{aligned}$$

with $\epsilon$ being the single molecule brightness and *N* being the average number of molecules in the observation volume. The coefficient $\gamma _r$ is generally defined as [10]

5 $$\begin{aligned} \gamma _r = \frac{\int _V (\overline{PSF}(\mathbf {r}))^r\,\mathrm {d}\mathbf {r}}{\int _V \overline{PSF}(\mathbf {r})\,\mathrm {d}\mathbf {r}}. \end{aligned}$$

In case of $r = 2$ and by approximating the normalized *point spread function* $\overline{PSF}$ by a Gaussian beam profile

6 $$\begin{aligned} \overline{PSF}(\mathbf {r}) = \exp {\left( -2\frac{(x^2+y^2)}{r_0^2}-2\frac{z^2}{z_0}\right) }, \end{aligned}$$

$$\gamma _2$$ becomes

7 $$\begin{aligned} \gamma _2 = \frac{1}{2\sqrt{2}}. \end{aligned}$$

Now we abandon the limitation to short sampling times and consider the case of arbitrarily large sampling times for the *Q* parameter.

8 $$\begin{aligned} Q(T)&= \frac{\kappa _{2}(T)}{\kappa _{1}(T)} \end{aligned}$$

The sampling time-dependent first intensity cumulant $\kappa _{1}(T)$ can be defined as follows [12, 14]:

9 $$\begin{aligned} \kappa _{1}(T) = \epsilon \,N = \mu _0\,T\,N \end{aligned}$$

with $\mu _0 = \frac{\epsilon }{T}$ being the count rate of a single molecule. The sampling time-dependent second intensity cumulant $\kappa _{2}(T)$ is given by [12, 14]

10 $$\begin{aligned} \kappa _{2}(T) = \gamma _2\,\mu _0^2\,T^2\,N\,\Gamma _{\text {diff}}(T). \end{aligned}$$

The dimensionless binning function $\Gamma _{\text {diff}}$ describes the dependence of the second intensity cumulant on the data sampling time and is defined as [14]

11 $$\begin{aligned} \Gamma _{\text {diff}}(T) = \frac{2}{T^2} \int _0^T (T - \tau )\, g(\tau )\, \mathrm {d}\tau . \end{aligned}$$

For a three-dimensional Gaussian *PSF*, the correlation function is

12 $$\begin{aligned} g_{\text {3DG}}(\tau ) = \left[ \left( 1+ \frac{\tau }{\tau _D}\right) \sqrt{1+ \frac{\tau }{r^2\tau _D}}\right] ^{-1}, \end{aligned}$$

with $r^2 = z_0^2/r_0^2$ being the ratio of the axial $z_0$ and lateral $r_0$ expansion of the Gaussian *PSF* Eq. 6. Solving Eq. 11 for $g_{\text {3DG}}$ yields [13, 15]:

13 $$\begin{aligned} \Gamma _{\text {diff,3DG}}(T) = {\left\{ \begin{array}{ll} \frac{8}{\alpha ^2} \left( \frac{\alpha }{2} - \sqrt{1+ \alpha } +1 \right) &{}\text {for } r = 1, \\ \frac{4}{\alpha ^2 \beta } \Bigg [ \frac{ \beta (1+\alpha )}{\sqrt{1-\beta }} artanh{\left( \sqrt{1-\beta } \frac{(\sqrt{1+ \alpha \,\beta } - 1)}{(\beta + \sqrt{1+ \alpha \, \beta } - 1)}\right) } - \sqrt{1 + \alpha \,\beta } +1\Bigg ] &{}\text {for } r > 1. \end{array}\right. } \end{aligned}$$

with the dimensionless sampling time $\alpha = T/\tau _D$ and $\beta = r^{-2} = r_0^2/z_0^2$. Inserting Eqs. 9, 10 and 13 into Eq. 8 yields the Q parameter as a function of the sampling time *T*.

14 $$\begin{aligned} Q_{\text {3DG}}(T) = \gamma _2\,\mu _0\,T\,\Gamma _{\text {diff,3DG}}(T) \end{aligned}$$

**Fig. 1:**
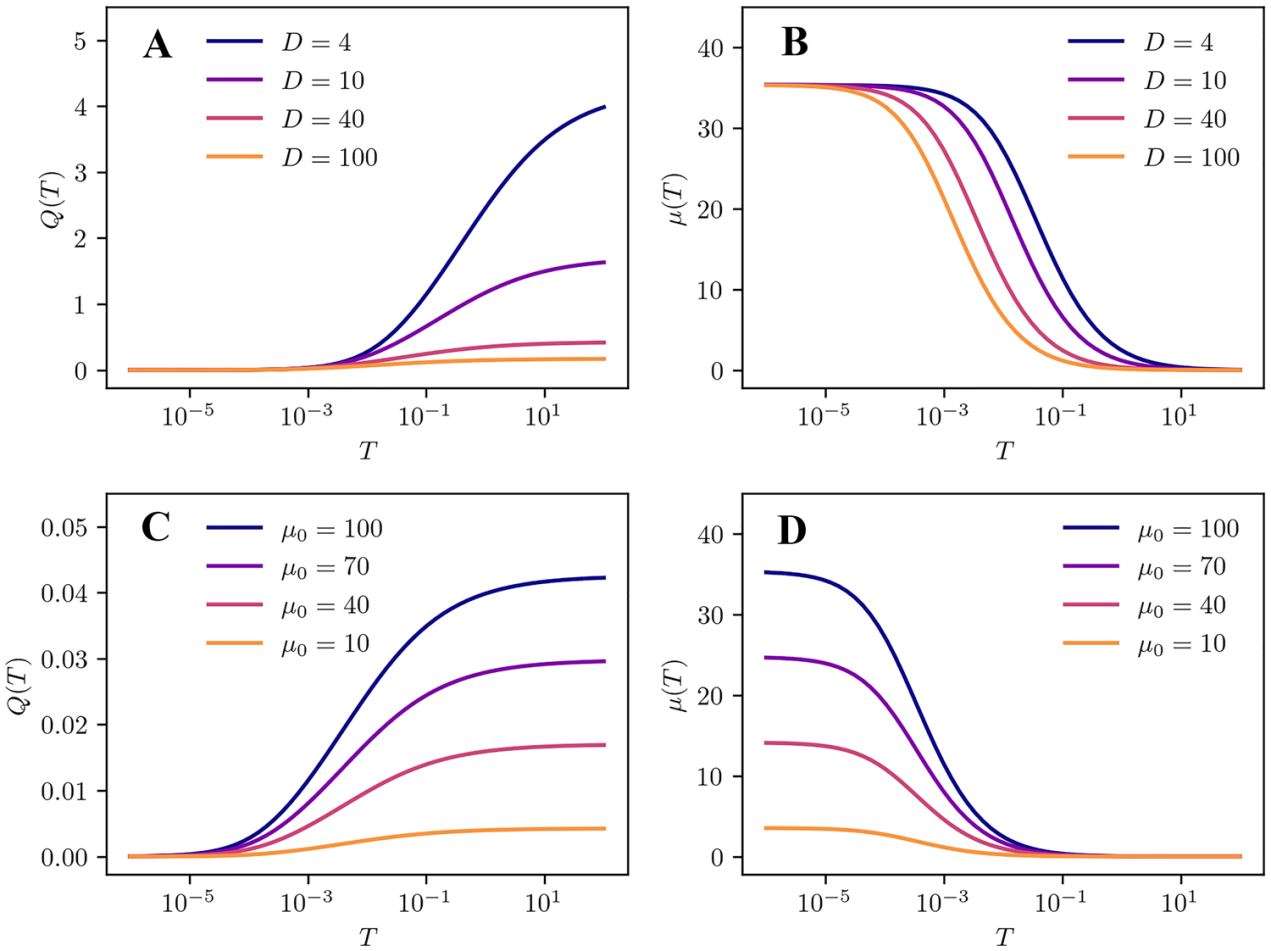
Comparison of Eqs. 14 and 15. The left side of the subplots shows Mandel’s Q parameter *Q*(*T*) and the right side displays the mSMR $\mu (T)$. **A ** and **B** Varying diffusion coefficient *D*. The rate of the single molecule brightness is constant at $\mu _0 = {100}{kCps}$. **C** and **D** Varying single molecule brightness rates $\mu _0$. The diffusion coefficient is constant at $D = {40}{\mu m^2/s}$. The geometric parameter is $r = 10$ in all graphs

An expression is obtained which for given geometric parameters *r* depends solely on the rate of the single molecule brightness $\mu _0$ and the mean diffusion time $\tau _D$. Figure 1 shows on the left side the curves of Eq. 14. By dividing Eq. 14 by the sampling time *T*, we obtain the mean single molecule rate (mSMR):

15 $$\begin{aligned} \mu _{\text {3DG}}(T) = \frac{Q(T) }{T} = \gamma _2\,\mu _0\,\Gamma _{\text {diff,3DG}} \end{aligned}$$

With the mSMR the influence of the diffusion time $\tau _D$ becomes immediately apparent from the graphs in subplot B and D. In these plots, the curve progression is strongly reminiscent of the curve progression in fluorescence correlation spectroscopy analyses. But instead of the mean particle count, the mean rate of the single molecule brightness rate is now determined via the amplitude of the curve.

### Measurement Artefacts

In fluorescence fluctuation spectroscopy, several interfering factors occur. We will address the most common artefacts afterpulsing, detector dead time and background noise and present correction terms for the mSMR.

#### Afterpulsing and Detector Dead Time

Photon counting devices are not perfect and are themselves sources of error. Afterpulsing and detector dead time are the most common artefacts in FFS experiments that use single-photon avalanche diodes. Afterpulsing affects all modern photon counting devices. Whenever a signal is triggered at the detector, there is a certain probability that another spurious photoelectron event is triggered. The probability of an afterpulsing event is detector-specific and decreases rapidly over time [18]. Typical probabilities for the occurrence of afterpulsing are in the range of 1% and have a nanosecond to microsecond delay to the true signal [21]. It is important to note that an afterpulsing event can cause further afterpulsing events, which are referred to as second or third order afterpulsing. Afterpulsing increases Mandel’s Q parameter [22] and should therefore be considered in an analysis based on Mandel’s Q parameter. The second artefact is detector dead time. After triggering an electron avalanche, the detector needs a short periode of time to return to its original base state. This time is called the detector dead time. During the dead time no further photon events can be detected. The detector is therefore blind for a short time. Typical detector dead times are in the nanosecond range. Dead time reduces Mandel’s Q parameter, which means that the measured value of the Q parameter is smaller than the true value [22]. To keep the influence of detector dead time as low as possible, the detector should generally not be operated at too high count rates. To account for afterpulsing and detector dead time, the photocounting moments from the experiment $\langle k^n \rangle _m$ can be corrected. To facilitate further analyses, it is convenient to correct the measured moments directly. The first two moments $\langle k \rangle$ and $\langle k^2 \rangle$ corrected for first order afterpulsing and detector dead time is given by [23]:

16 $$\begin{aligned} \langle k \rangle&= \langle k \rangle _m (1 - P_A - \delta ) + \delta \langle k^2 \rangle _m, \end{aligned}$$

17 $$\begin{aligned} \langle k^2 \rangle&= \langle k^2 \rangle _m (1-2 P_A - 3 \delta ) + 2 \delta \langle k^3 \rangle _m + (\delta - P_A)\langle k \rangle _m, \end{aligned}$$

where $P_A$ is the afterpulsing probability of the detector and $\delta$ is the dimensionless dead time given by $\delta = \frac{t_{\text {dead}}}{T}$ with the detector-specific dead time $t_{\text {dead}}$. The above equations are only valid for the case that $\langle k \rangle \delta \ll 1$. If this condition is not met, the correction of the first two moments of a fluorescence trace is erroneous.

#### Background Noise

In addition to detector artifacts, the background noise of the measurements has also an effect on the mSMR data. Background noise typically lowers the mSMR data and causes an underestimation ot the single molecule brightness rate. This effect is also known in fluorescence correlation spectroscopy, where a decrease in the S/N ratio affects the average number of particles $\langle N \rangle$ in the detection volume. To compensate for this effect a correction term $\chi ^2$ is commonly used [24, 25].

18 $$\begin{aligned} \chi ^2 = \left( 1+ \frac{\langle b \rangle }{\langle k \rangle - \langle b \rangle }\right) ^2, \end{aligned}$$

with the background noise $\langle b \rangle$. We can adapt this term for the rate of the single molecule brightness as follows:

19 $$\begin{aligned} \begin{aligned} \mu _{0, corr}&= \frac{\langle k\rangle - \langle b\rangle }{\langle N\rangle _{corr}} = \frac{\langle k\rangle - \langle b\rangle }{\langle N\rangle } \chi ^2\\&=\left( 1-\frac{ \langle b\rangle }{ \langle k\rangle }\right) ^{-1} \mu _0. \end{aligned} \end{aligned}$$

The term is suitable to correct $\mu _0$ from the experiment. The background noise is determined for the correction via a blank measurement. However, sometimes it is more practical to correct the mSMR curves directly. For this we can apply the background correction to the sampling time-dependent model.

20 $$\begin{aligned} \mu _{corr} (T) = \gamma _2 \mu _0 \Gamma _{\text {diff}} \left( 1-\frac{\langle b \rangle }{\langle k \rangle } \right) ^{-1} \end{aligned}$$

### Photokinetic Effects

While diffusive processes occur at comparatively large sampling times, there are further photokinetic effects that occur at very short sampling times. Frequently observed phenomena are the occurrence of triplet states, rotational diffusion and for some dyes isomerization effects, which influence the emission characteristics of the particles under study [26]. Since we use very small laser powers in this study, triplet effects can be neglected. We also work with time resolutions of $\mu s$. In contrast, rotational diffusion takes place in nanoseconds and cannot be resolved in our setting. On the other hand, the influence of isomerization is not negligible for labeled DNA polymers. Commonly used fluorescence dyes for RNA/DNA labeling (e.g. RiboGreen or PicoGreen) belong to the group of cyanine dyes. These are known to exhibit cis/trans isomerization, which leads to blinking upon fluorescence excitation [27]. In the simplest case, isomerization is a two-state system that switches back and forth between a bright and a dark state. Assuming that the isomerization takes place on much shorter time scales than the diffusion process, the diffusion process can be regarded as stationary relative to isomerization. That allows consideration this process independently. The isomerization term for a simple on/off system has the same shape as the term for triplet effects and is given by [28]:

21 $$\begin{aligned} g_{\text {iso}}(t) =1 + \frac{ F }{1-F }\exp ^{-\frac{t}{\tau _F}}. \end{aligned}$$

*F* is the fraction of fluorophores in a dark state and $\tau _F$ is the sum of the switching rates $k_{\text {on}}$ and $k_{\text {off}}$. For the mSMR the additional term is handled in the same way as the diffusion term [13]. Thus, inserting Eq. 21 into Eq. 11 and integrating over the sampling time yields

22 $$\begin{aligned} \Gamma _{\text {iso}}(T)&= \frac{2}{T^2} \int _0^T (T - \tau )\, g_{\text {trip}}(\tau )\, \mathrm {d}\tau \nonumber \\&=1 +\frac{2}{T^2} \frac{ F\, \tau _F\, T - (1 - \exp ^{-\frac{T}{\tau _F}}) F\, \tau _F^2)}{(1-F)}. \end{aligned}$$

This results in the entire expression for $Q(T)$:

23 $$\begin{aligned} Q(T) = \gamma _2\,\mu _0\,T\,\Gamma _{\text {diff}}(T)\,\Gamma _{\text {iso}}(T), \end{aligned}$$

and for the sampling time-dependent mSMR $\mu (T)$:

24 $$\begin{aligned} \mu (T) = \gamma _2\,\mu _0\,\Gamma _{\text {diff}}(T)\,\Gamma _{\text {iso}}(T). \end{aligned}$$

A simple isomerization model for fluorescence correlation spectroscopy analysis has been used to study the blinking of GFP [26] and the cis-trans isomerization of the fluorescent cyanine dye Cy5 [29]. In reality, *M* fluorophores bind to a DNA polymer. This is a *finite birth and death process* with $M + 1$ states from completely dark to all fluorophores are emitting. For fluorescence correlation spectroscopy analysis such a system can be modeled by a system of coupled differencial equations [28]. However, such a system can no longer be represented analytically and consists of a linear superposition of exponential functions with the eigenvalues as decay constants.

25 $$\begin{aligned} g_{\text {iso}}(t) = \sum _{i=0}^M b_i\,\exp {(\lambda _i\,t)} \end{aligned}$$

In practice, the simplified model of a binary on/off isomerization is a sufficiently good description of the experimental data. Therefore, we will use the simplified, though physically inaccurate, assumption of a binary on/off isomerization in our modeling.

## Materials and Methods

### Monte Carlo Simulation

In order to investigate the models without noise sources (detector artefacts, isomerization effects), the diffusion of particles is modeled in a Monte Carlo simulation. The simulation volume is defined with $12 \times$ the axial and longitudinal extension $z_0$ and $r_0$ of the detection volume. The minimum step size $\epsilon$ is determined by the fastest simulated species and is limited to a maximum of 20nm to ensure the validity of a *Wiener process* to describe a random walk of diffusing species. Using the *Einstein-Smolouchowski relationship*, the time slice $\Delta t$ for the simulation of a 3D diffusion is given by

26 $$\begin{aligned} \Delta t = \frac{\epsilon ^2}{6D}. \end{aligned}$$

The random direction of motion of each particle is equally distributed in the 6 spatial directions. A Gaussian profile is used as molecular detection efficiency profile (see Eq. 6). The normalized Gaussian profile is multiplied by a factor $\phi _0$ which summarizes all optical properties of the simulated particles (e.g. quantum efficiency, cross section, excitation intensity), thus giving us:

27 $$\begin{aligned} \phi (\mathbf {r}) = \phi _0\,\overline{PSF}(\mathbf {r}). \end{aligned}$$

The number of photons emitted by each particle at location $\mathbf {r}$ is modeled by a Poisson distribution

28 $$\begin{aligned} \text {Poi}(\phi (\mathbf {r}), k) = \frac{(\phi (\mathbf {r}))^k}{k!} e^{-\phi (\mathbf {r})}. \end{aligned}$$

The Poisson distribution returns the probability to detect *k* photons from a simulated particle at position $\mathbf {r}$ with a corresponding local photon count $\phi (\mathbf {r})$. At the beginning of each time slice the position of all simulated particles is updated by a random motion in *x*, *y* or *z* direction, whereas the step size of each particle depends on its diffusion coefficient. After updating the position of all particles, Eq. 28 is used to calculate the total number of emitted photons for all particles. The successive calculation of the total number of photons of all simulated particles for each time slice yields the fluorescence trace. The simulation can easily be extended to include additional noise factors such as Poissonian noise. The simulated fluorescence trace is analyzed using Eq. 32 for increasing sampling times without correcting for detector artefacts. The fitting of the data is done via Eq. 15.

### Instrumentation

**Fig. 2:**
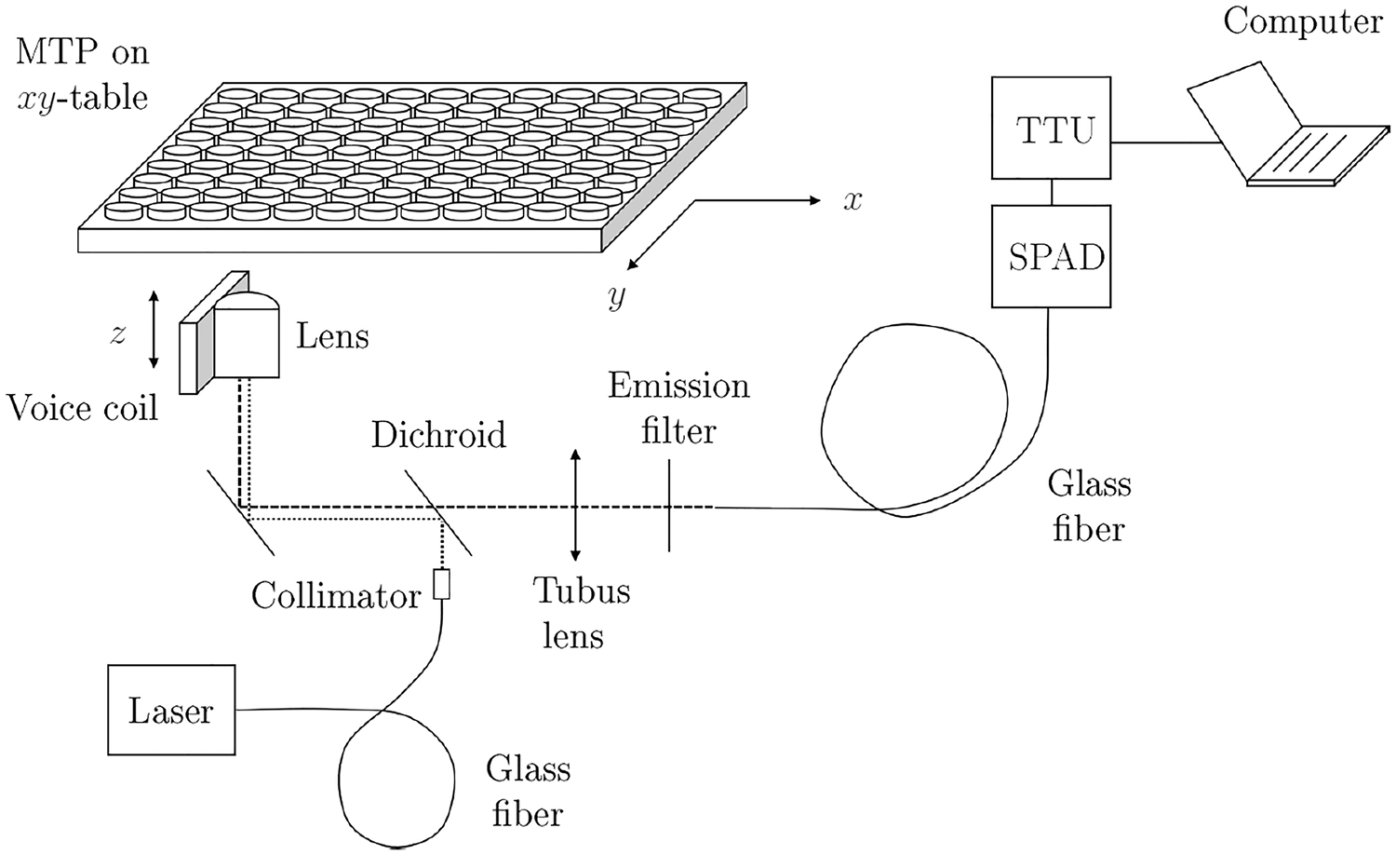
Laboratory setup of the confocal plate reader for the measurements in our study. The wells of a microtiter plate are scanned in sequence via an *xy* stage, with a *z*-adjustor moving the objective lens to the optimal focal point for the measurement. The excitation beam is coupled into the system via a fiber. The fluorescence photons emitted from the sample are focused via a tube lens onto the detection fiber, where the incident photons are detected by a SPAD and subsequently processed by a time tagger card

The measuring system used is a home-built confocal plate reader. A schematic representation of the system used is shown in Fig. 2. A fiber-coupled laser (488nm, Laser2000, France) serves as a photon source.

The laser beam is directed via a dichroic mirror (LP500) into a microscope objective (Neofluar, 63x/0.75, LD, Zeiss, Germany) and focused on the sample in a microtiter plate with transparent bottom. A part of the emitted fluorescence photons is collected by the objective and can now pass the dichroic mirror due to the *Stokes shift*. An emission filter (535/50) filters out any remaining residual excitation light. An optical fiber (50$\mu m$, Thorlabs, Germany) serves as a pinhole and transmits the measurement signal to a single-photon avalanche diode (PDM, Micro Photon Devices, Italy) with a dead time of 70*ns* and an afterpulsing probability of $< {1}\%$ (according to the manufacturer’s specifications). The TTL signal from the diode is processed by a time tagger card (Time Tagger 20, Swabian Instruments, Germany) and sent to the computer where data processing takes place. The reading of the wells is automated. Via an *xy*-table (Jüke, Germany) the wells of the plate are scanned successively. An autofocus routine automatically finds the sharpest measuring plane by moving the objective lens step by step in *z*-direction via a voice coil (PI, Germany). Measurement data are managed and hardware is controled by a software developed by the authors of this study. The objective lens used in the setup is a long-distance air lens instead of an oil or water immersion lens. We have shown the feasibility of using a long-distance lens for FFS experiments in other study [30]. The main advantage is that the setup is less costly and easier to automate.

### Data Processing

The measurement data as well as the data generated by the Monte Carlo simulations have to be processed to derive the sampling time-dependent moments. To calculate the mSMR from a given fluorescence trace over a total observation time $T_{\text {obs}}$, we proceed as follows. The minimum sampling time of the fluorescence traces of the FFS measurement is given by the measurement setup and the specifications during data acquisition. We divide the fluorescence trace of *n* entries into all possible equidistant sampling intervals with $T = t_i - t_{i-1}$ with $i = 1, 2, 3, \dots , N$, also referred to as sampling time. We accomplish this by finding all integer divisors *N* for the length *n* of the original fluorescence trace. We then perform a rebinning of the fluorescence trace, where the number of combined bins corresponds to the sampling time. A quick way of binning an array is via matrix multiplication. We illustrate this with the example of a fourfold binning. We start with a fluorescence trace with *n* entries and an original binning time of 1 $\times 10^{-6}$s

29 $$\begin{aligned} F_{n \times 1} = \begin{bmatrix} a_{1} \\ \vdots \\ a_{n} \end{bmatrix} \end{aligned}$$

By a reshape, the $n \times 1$ matrix of the fluorescence trace is converted into a $n/4 \times 4$ matrix. For clearity, we keep the original indexing of the entries.

30 $$\begin{aligned} F_{\frac{n}{4} \times 4} = \begin{bmatrix} a_1 &{} a_2 &{} a_3 &{} a_4\\ \vdots &{} \vdots &{} \vdots &{} \vdots \\ a_{n-3} &{} a_{n-2} &{} a_{n-1} &{} a_{n} \end{bmatrix} \end{aligned}$$

We multiply the matrix with a $4 \times 1$ matrix containing only ones values. The result is the binned $n/4 \times 1$ matrix with a sampling time of 4 $\times 10^{-6}$s.

31 $$\begin{aligned} F_{\frac{n}{4} \times 1} = F_{\frac{n}{4} \times 4} \times \begin{bmatrix} 1&1&1&1 \end{bmatrix}^{\intercal } = \begin{bmatrix} b_1 \\ \vdots \\ b_{n/4} \end{bmatrix} \end{aligned}$$

The rebinning is repeated for all integer divisors of *n*. For each rebinned fluorescence trace, we calculate the associated first and second moments of the photon counts and finally get the sampling time-dependent *Q* parameter, which we normalize to the corresponding sampling time *T*:

32 $$\begin{aligned} \mu (T) = \frac{Q(T)}{T} = \frac{\langle k^2 \rangle _m(T) - \langle k \rangle _m^2 (T) - \langle k \rangle _m (T) }{T \langle k \rangle _m (T)}. \end{aligned}$$

To account for the effects of afterpulsing and detector dead time, the two measured moments $\langle k \rangle _m$ and $\langle k^2 \rangle _m$ are corrected via Eqs. 16 and 17 before they are inserted into the above equation. Subsequently, $\mu (T)$ is fitted with Eq. 15 using a least square non-linear fitting routine to derive the physical parameters.

### Sample Preparation and Data Acquisition

Double stranded DNA fragments of defined length (Nolimits DNA fragments, Thermofisher, USA) are used in the measurements. The dsDNA fragments (50bp, 100bp, 200bp, 300bp, 500bp, 700bp, 1000bp) and DNA library solutions are prepared in 25% DMSO and 75% water (vol/vol) and adjusted to the following concentrations: 0, 5, 10, 25, 50, 100, 150 and 200pg/$\mu l$. A 0.6x solution of the cyanine dye RiboGreen (997$\mu l$ TE-buffer and 3$\mu l$ RG stock solution) is used for labelling the DNA molecules. Equal volumes of DNA solution and 0.6x dye solution are mixed and equilibrated for 2*h*. RiboGreen stains all types of nucleic acids and has been shown in pre-tests to be very bright and to yield accurate results [30].

For the measurements, 40$\mu l$ of the samples are transferred into a 384 well microtiter plate ($\mu$Clear, non-binding, Greiner BioOne, Germany). The algorithm controling the measurement setup automatically finds the bottom side of the microtiter plate and performs the measurement according to the given parameters. For each well, five measurements are recorded at different locations. The measurement time for each point is 10s. The laser power is set to 2.5$\mu W$ or 10$\mu W$ (see captions below for details). The fluorescence traces are processed as described in section “Data Processing” and fitted with the model that includes the isomerization term (Eq. 24). The fit parameters of the five measurements in each well are averaged.

## Results and Discussion

### Simulations

**Fig. 3:**
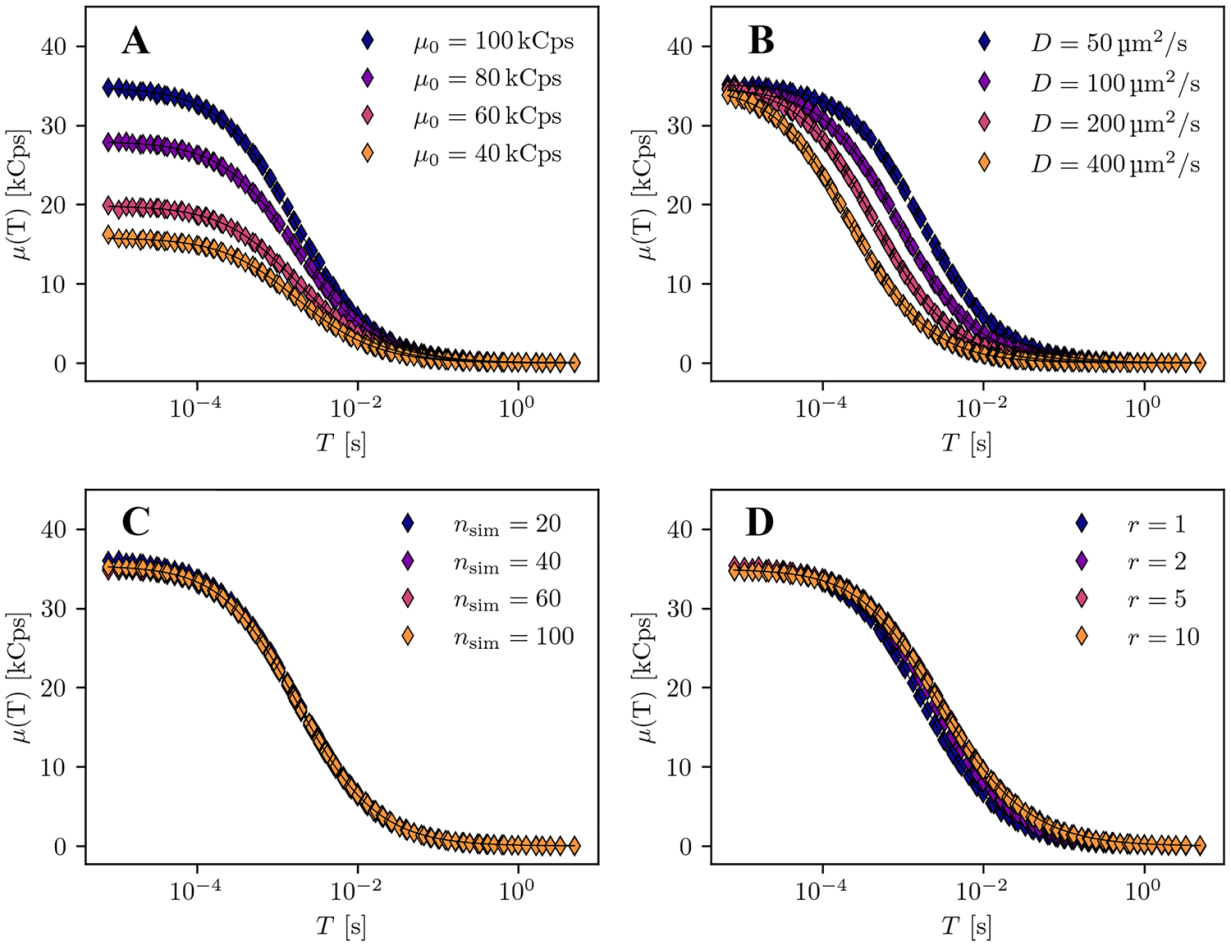
mSMR from simulated fluorescence traces. The general parameters are: $\omega _{xy} = {0.4}{um}$, simulation time $t = {10}{s}$. **A** Varying rate of single molecule brightness $\mu _0$, with constant geometric parameter $r = 1$, diffusion coefficient $D = {50}{\mu m^2/s}$ and particle number $n_{\text {sim}} = 20$. **B** Varying diffusion coefficients *D*, with constant geometric parameter $r = 1$, single molecule brightness rate $\mu _0 = {100}{kCps}$ and particle number $n_{\text {sim}} = 20$. **C** Varying particle numbers $n_{\text {sim}}=20$, with constant geometric parameter $r = 1$, diffusion coefficient $D = {50}{\mu m^2/s}$ and single molecule brightness rate $\mu _0 = {100}{kCps}$. **D** Varying geometric parameter *r*, with constant diffusion coefficient $D = {50}{\mu m^2/s}$, single molecule brightness rate $\mu _0 = {100}{kCps}$ and particle number $n_{\text {sim}} = 20$

The mSMR model is checked for plausibility using Monte Carlo simulation. This allows us to vary parameters that are difficult to change experimentally, such as the single molecule brightness rate or the geometric dimensions of the detection volume. In addition, disturbing experimental factors such as detector afterpulsing or dead time can be neglected in the simulation. All simulations are run five times for a measurement time of 10s. The lateral expansion of the confocal volume is $r_0 = {0.4}{\mu m}$. Figure 3 shows the results of the simulations. The data points are the average of the five individual simulation runs. The data are fitted with Eq. 15. Subplot A shows the curves of the mSMR $\mu (T)$ for different single molecule brightness rates ($\mu _0 = {40}{kCps}$, 60*kCps*, 80*kCps*, 100*kCps*). The diffusion coefficients and the mean particle number are constant ($D = {50}{\mu m^2/s}$ and $n_{\text {sim}} = 20$). The geometric parameter is $r = 1$. We see that the amplitude of the curves increases with increasing rates for the single molecule brightness. From the fits, we get the single molecule brightness rates with the corresponding standard diviations: $\mu _0 = 40.5\pm 0.6$, $60.3\pm 1.4$, $79.2 \pm 1.8$ and $98.4 \pm {2.6}{kCps}$. These results are in perfect agreement with the initial simulation parameters for $\mu _0$. Subplot B shows the curves of $\mu (T)$ for varying diffusion coefficients ($D = 50, 100, 200, {400}{\mu m^2/s}$). The rate of the single molecule brightness and the particle number are kept constant ($\mu _0 = {100}{kCps}$ and $N = 20$). The geometric parameter is $r = 1$. For decreasing diffusion coefficients, the curves shift towards larger sampling times. From the fits of the data we obtain the mean diffusion time $\tau _D$ that the particles need on average to pass through the detection volume. From the diffusion time, the diffusion coefficient is calculated using the following relationship:

33 $$\begin{aligned} D = \frac{r_{0}^2}{4 \tau _D}. \end{aligned}$$

The following diffusion coefficients and their standard deviations are derived from the fit of the simulated data: $D = 53 \pm 3$, $102 \pm 5$, $204 \pm 6$ and $407 \pm {16}{\mu ^2/s}$. Once again the results are in good agreement with the initial parameters of the simulation. Subplot C shows the results of the simulation for varying numbers of particles ($N = 20$, 40, 60 and 100) at a constant single molecule brightness rate ($\mu _0 = {100}{kCps}$) and constant diffusion coefficient ($D = {50}{\mu m^2/s}$). The geometric parameter is $r = 1$. As expected, the mSMR curves show no significant deviations from each other, since the single molecule brightness rate is the same in all simulations. Subplot D finally shows the data for varying geometric parameters. The axial expansion was gradually increased ($z_0 = 0.4$, 0.8, 2.0 and ${4.0}{\mu m}$) while the lateral expansion was kept constant ($r_0 = {0.4}{\mu m}$) resulting in $r = 1$, 2, 5 and 10. The other simulation parameters were held constant ($N = 20$, $\mu _0 = {100}{kCps}$ and $D = {50}{\mu m^2/s}$). As expected, the variation of the geometric parameter *r* shows no influence on the amplitude of the curves and thus on $\mu _0$. The sigmoidal decline of the data at larger sampling times, however, is influenced by the axial expansion. But the influence is very small. Between the curves of $r = 1$ and $r = 2$ there is still a clear difference. But the curves of $r = 5$ and $r = 10$ practically coincide. Thus, for large *r*, the sensitivity of the model to variations in *r* is very weak. For the fit of data from real measurements, we recommend not to leave the geometric parameter *r* freely adjustable, but to determine it via a precise calibration using a fluorescent dye of known concentration (e.g. Alexa 488), and considering *r* fixed for further experimental evaluations.

By systematically varying the molecular parameters in the Monte Carlo simulations, we demonstrated that the mSMR model can reliably reproduce the initial simulation parameters and behaves according to the theoretical expectations. However, the model is very insensitive to variations of the geometric parameter *r*. We recommend to determine the geometric parameter by calibration measurements for the confocal setup and to include it as a fixed parameter in the non-linear fit of the model to the data points.

### Experiments

After checking the model with simulated data, we now test our model against data from real measurements. We do this with measurements on double-stranded DNA mixtures of known composition.

#### Measurements Artefacts

**Fig. 4:**
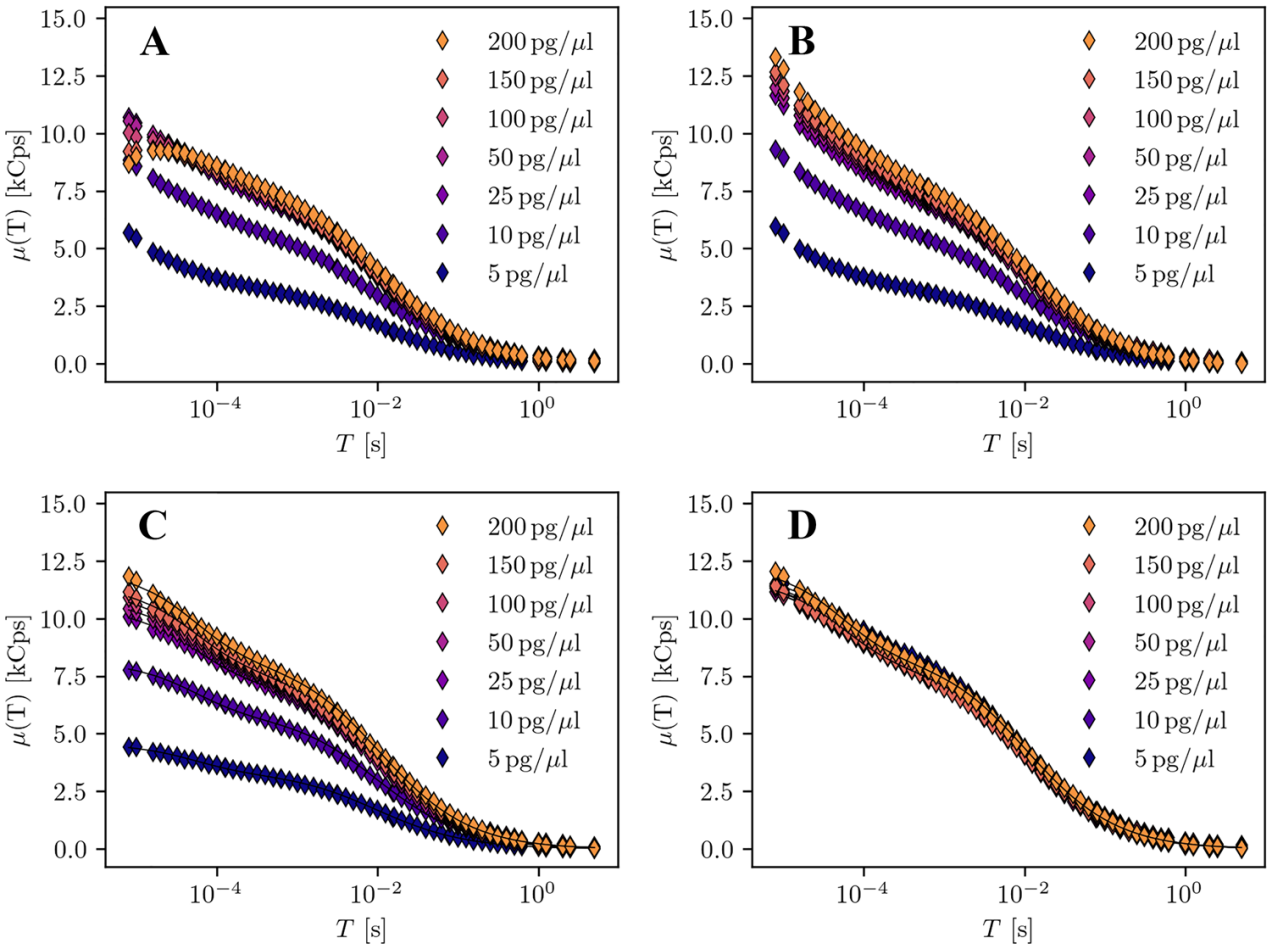
Stepwise correction of the mSMR using the example of a 100bp DNA dilution series labeled with RiboGreen at 10$\mu W$ excitation power. **A** Uncorrected data from the measurement. **B** Data corrected for detector dead time. **C** Data corrected for detector dead time and afterpulsing. Fit of the data points with Eq. 24. **D** Data corrected for detector dead time and afterpulsing with additional correction for the background noise of the measurement using Eq. 19. Fit of the data via Eq. 24

In the analysis of real measurements, detector artefacts play an important role, especially for short sampling times. We focus on afterpulsing and detector dead time, the two most important artefacts, and demonstrate their effects in the evaluation of a 100bp DNA mixture. Additionally, we take into account the influence of background noise on the mSMR model. The measurements were conducted with an excitation power of 10$\mu W$ to make the effects more prominent. In later measurements we will work with somewhat lower excitation powers. Figure 4 shows the stepwise correction of the detector artefacts in the mSMR. We start in Subplot A with the raw mSMRs of a dilution series. It can be seen that the amplitudes of the mSMR curves differ, although the molecules studied are the same for all concentration steps. This difference is caused by background noise. In addition, it is noticeable that at short sampling times the data series of high concentration show a slightly deviating course from the other data series. This effect is caused by detector dead time. At higher laser powers and higher concentrations, this effect starts to dominate and prevents, without correction, a meaningful evaluation. We start with correcting $\mu (T)$ for detector dead time using Eq. 17 with a dead time of $t_{\text {dead}} = {70}{ns}$. Subplot B shows the detector dead time corrected results. The characteristic deviations at small sampling times between the data series have disappeared. Instead, there is now a slight exponential increase of $\mu (T)$ at short sampling times in all data sets. This overestimation of the amplitude is caused by afterpulsing. We now correct the data sets for afterpulsing by applying Eq. 17 with an afterpulsing probability of $P_{A} = 0.006$. In subplot C we see that the exponential character of the mSMR curves disappeared at short sampling times. The corrected data can be accurately fitted with Eq. 24, which takes a binary on/off isomerization into account. The remaining deviations in the amplitudes are caused by background noise, which has a much stronger influence on the curves at low concentrations than at high concentrations. We correct for this effect using Eq. 20. We determined the amount of background noise with a blank measurement. Subplot D finally shows the $\mu (T)$ curves completely corrected for afterpulsing, detector dead time and background noise. The data points are now in good alignment for all concentration steps. We perform these correction steps for all collected data sets in this study.

#### DNA Mixtures

**Fig. 5:**
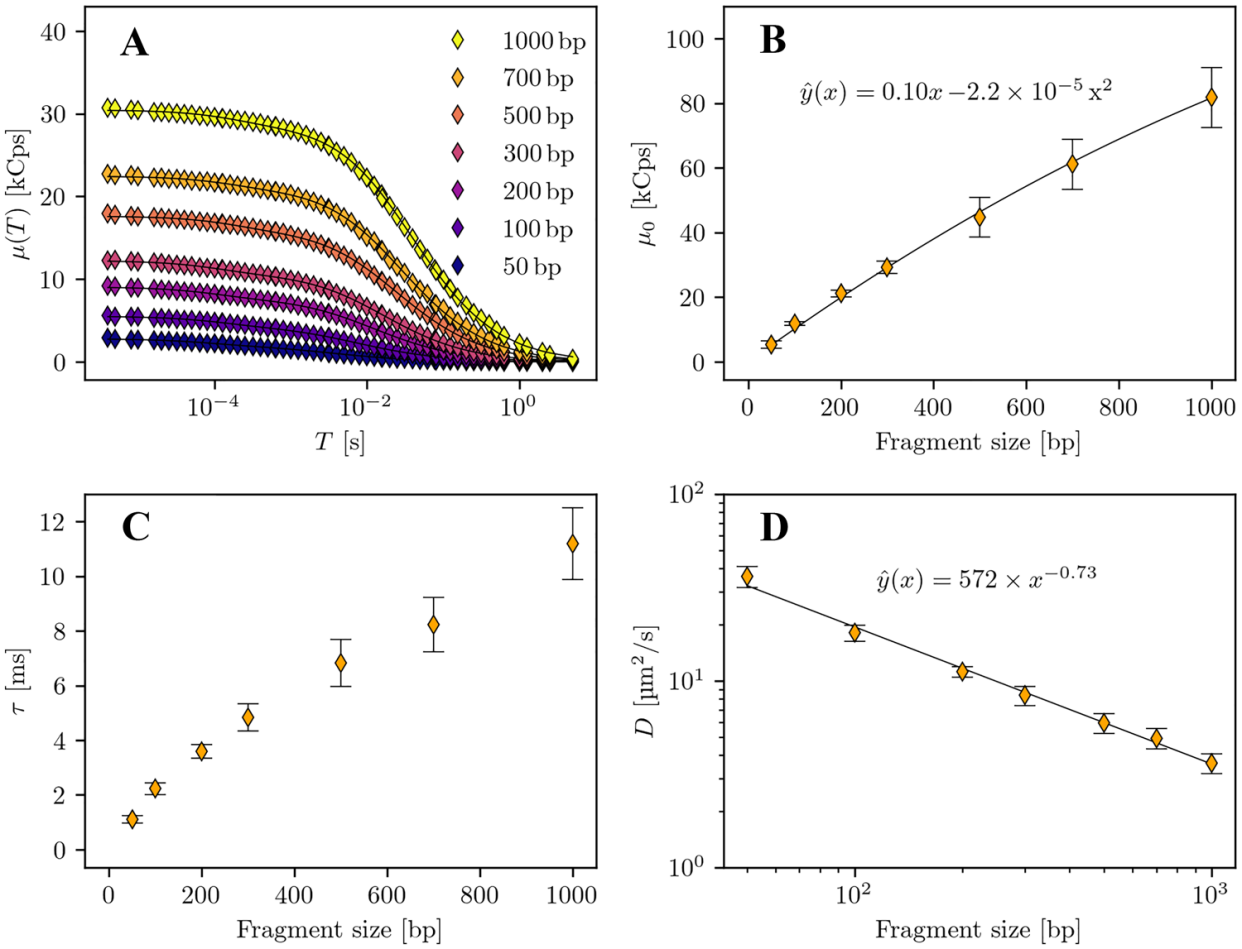
Summary of the results from the analysis of DNA solutions consisting of defined fragment lengths with concentration steps of 5, 10, 25, 50, 100, 150, 200pg $\mu l$. The excitation power is ${2.5}{\mu W}$. **A** Averaged mSMR curves of the DNA dilution series. **B** Averaged corrected $\mu _0$ with standard deviations of the DNA dilution series. The data points are fitted with a polynomial of second order ($f(x) = a\,x^2 + b\,x + c$). **C** Averaged diffusion times $\tau$ with standard deviations of the DNA dilution series. **D** Averaged diffusion coefficients *D* with standard deviations of the DNA dilution series in a double logarithmic presentation. The data points are fitted using a power law ($f(x) = A \times x^B$)

In the following, we study the mSMR model with DNA solutions of defined fragment lengths. Dilution series are prepared and evaluated for each fragment length. The studied concentrations are 5, 10, 25, 50, 100, 150, 200 pg/$\mu l$. The laser power used is set to 2.5$\mu W$ to minimize detector artefacts and to keep photobleaching as low as possible. We correct all data sets for measurement artifacts. A summary of the results is shown in Fig. 5. Subplot A shows the averaged $\mu (T)$ curves over all studied concentration steps. The increase in amplitude and the shift of the data series towards larger sampling times with increasing fragment lengths is obvious. All data series can be reliably fitted with Eq. 24 with isomerization term. Subplot B displays the averaged single molecule brightness rates of each dilution series. With increasing fragment length also $\mu _0$ increases. The increase in brightness is to be expected as more marker molecules can bind to larger DNA fragments and contribute to the overall brightness of the molecule. The data points can be fitted with a polynomial of second order. The deviation from an ideal straight line is presumably due to size effects of the DNA molecules when traversing the detection volume. However, despite the low excitation powers, increasing photon bleaching effects for larger molecules cannot ruled out either [30]. Subplot C shows the average diffusion times of the dilution series. With increasing fragment size, the diffusion time also increases. The diffusion coefficients of the DNA fragment solutions are determined from the mean diffusion time using Eq. 33 with a lateral expansion of $r_0 = {0.40}{\mu m}$ from a calibration measurement. The diffusion coefficients are shown in Subplot D in a double-logarithmic representation. The data points can be described by a power law. The fit yields an exponent of $B = -0.73 \pm 0.03\,\,\text {(standard error)}$, which is in almost perfect agreement with previously reported values in literature ($B = -0.72$ [31]). This indicates that the measurements are in line with the expectations from the literature.

**Fig. 6:**
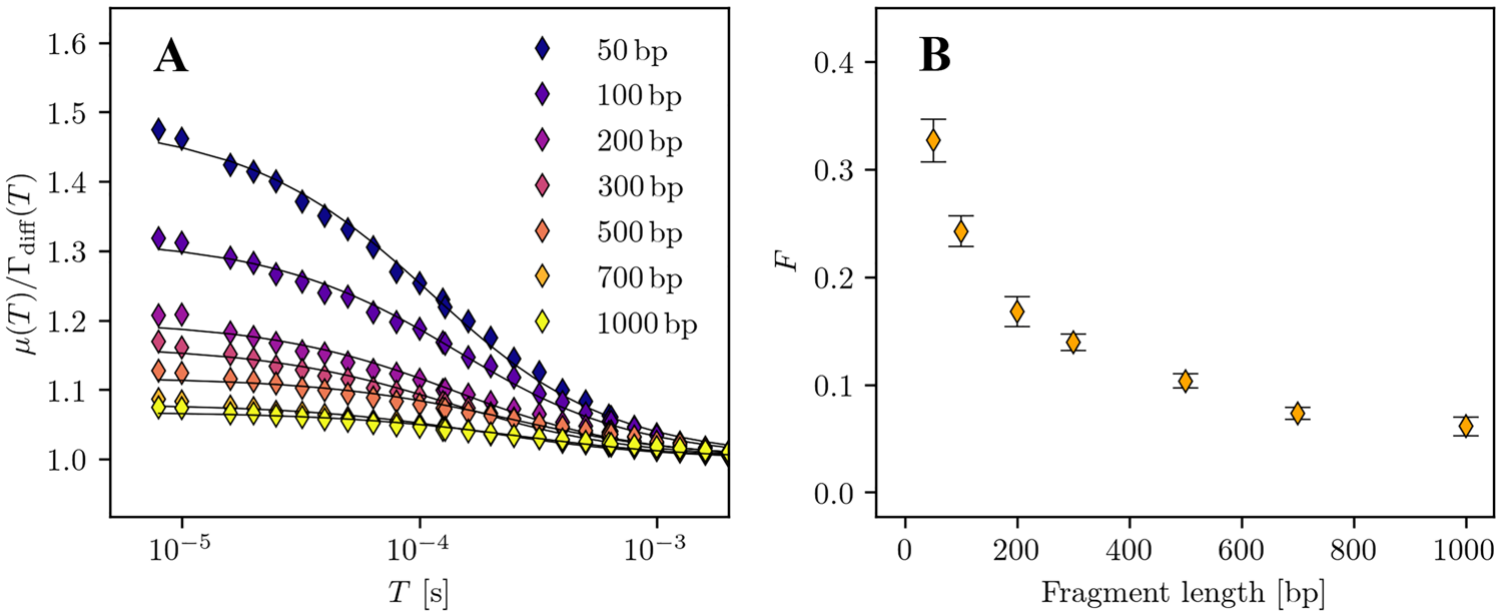
Photokinetic effects in the mSMR curves of DNA measurements. **A** Division of the mSMR curves by the diffusive part $\Gamma _{\text {diff}}$ of the mSMR model. **B** The mean proportions *F* with standard deviations from the isomerization term for the DNA mixtures

We will look a little closer at the data set and focus on isomerization effects at short sampling times. For this, we use the averaged mSMR curves from Fig. 5, subplot A. Since the measurements were taken at only 2.5$\mu W$ excitaion power, we initially assume that triplet states can be neglected and we can focus entirely on isomerization. We divide the mSMR curves by the diffusive fraction $\Gamma _{\text {diff}}$ of the fitted model, thereby isolating the fraction of isomerization $\Gamma _{\text {diff}}$ of the model at short sampling times. Figure 6, subplot A shows the results of this procedure.

We focus on sampling times from microseconds to milliseconds. The amplitudes of the isomerization fraction decrease significantly for longer fragment lengths and are practically negligible for 1000bp DNA fragments. We are reasonably accurate in fitting the isomerization fractions to the isomerization model Eq. 22, as indicated by the continuous graphs plotted. The isomerization fractions obtained from the fit are shown with their associated standard deviations in subplot B. The course of the data points is reminiscent of an exponential decay that asymptotically approaches a threshold. In the isomerization model, *F* describes the fraction of molecules that are in a dark state and cannot emit photons. This therefore means that for large DNA fragments, fewer molecules are in a dark state, which immediatelly makes sense since more dye molecules bind to them, reducing the probability of a completely dark state for the molecule. Although we employ a simplified on/off isomerization model, we can still accurately describe the mSMR curves at short sampling times.

## Conclusion

In this paper we presented the evaluation of fluorescence fluctuation experiments based on Mandel’s Q parameter for increasing sampling times, making use of the concept of factorial cumulants for larger sampling times, as used in FIMDA and TIFCA. By relating the Q parameter to the sampling time, we obtain the mean single molecule rate (mSMR), from which the characteristic diffusion properties of the sample under study can be directly interpreted visually, analogous to fluorescence correlation spectroscopy. The concept of the mSMR can be extended to include other photokinetic effects such as triplet state and isomerization. Our model is suitable for single photon excitation and a single detection channel. First, we demonstrated the functionality of the mSMR model by analyzing Monte Carlo-simulated fluorescence traces. The simulation allows to vary the input parameters systematically, and we could show that the model can reproduce the changes in these parameters. In a second step, real measurements were evaluated. Since real measurements include artefacts, especially afterpulsing, detector dead time and background noise, we presented correction mechanisms to account for these effects. Using the example of measurements on a dilution series of 100bp DNA solution, we demonstrated the individual disturbances introduced by the measurement artefacts and corrected them one by one. This enabled us to bring the measurement results of the DNA dilution series into perfect alignment. We then evaluated systematic measurements of DNA dilution series with increasing defined fragment lengths. The mSMR showed that the single molecule brightness rate increases for larger DNA fragments. This is in line with our expectations, as more dye molecules can bind to larger fragments. We also derived diffusion coefficients for the individual DNA mixtures from the mean diffusion times: The results are in line with data reported in the literature. The more detailed study of the isomerization in the DNA measurements showed that the mSMR can describe the measurement data very accurately, especially at short sampling times. We could observe how the fraction of labeled DNA molecules in a complete dark state decreases for large DNA fragments. This decrease seems to follow an exponential decay. This observation is plausible since RiboGreen is a cyanine dye and shows cis/trans isomerization. This means that it alternates between a bright fluorescent and a dark state. Since longer fragments have more dye molecules bound, the probability for a complete dark state of the dyed molecule decreases accordingly.

In further studies, we plan to go into more detail about the photokinetic effects in mSMR and plan to directly compare fluorescence correlation spectroscopy and mSMR. Initial observations suggest that mSMR may have an advantage here. Interesting future applications for the mSMR could be the analysis of in vivo hybridization experiments, e.g., the binding of two or more fluorescently labeled probes to a complementary target sequence could be studied in real time by mSMR, which should allow sequence-specific detection of nucleic acids directly in a biological system. Another application could be the temporal study of protein agglomeration in the cell membrane. Fluorescently labeled proteins diffusing into or along the cellular membrane could be observed by changes in diffusion times or single molecule brightness rate during agglomeration. In the latter case, we need to consider diffusion in 2D for the mSMR and need to adapt the model accordingly.

We believe that mSMR represents a substantial enrichment of fluorescence fluctuation methods. The mSMR can be calculated very quickly and evaluated analogously to the FCS. In addition, the ability to correct detector artifacts is a valuable advantage over the standard method of fluorescence correlation spectroscopy. We are convinced that our method can be an alternative to fluorescence correlation spectroscopy.

## Data Availability

The datasets generated during and/or analyzed during the current study are available via a public repository: 10.5281/zenodo.5148018.

## Code Availability

Minimal examples for data processing are available from the corresponding author on reasonable request.
